# Plant invasion: Another threat to the São Paulo Marsh Antwren (*Formicivora paludicola*), a species on the verge of extinction

**DOI:** 10.1371/journal.pone.0189465

**Published:** 2017-12-27

**Authors:** Glaucia Del-Rio, Marco Antonio Rêgo, Luís Fábio Silveira, Akira Itoh

**Affiliations:** 1 Departamento de Zoologia, Instituto de Biociências, Universidade de São Paulo, São Paulo, São Paulo, Brazil; 2 Seção de Aves, Museu de Zoologia da Universidade de São Paulo, São Paulo, São Paulo, Brazil; 3 Division of Biology & Geosciences, Osaka City University, Sugimoto, Sumiyoshi-ku, Osaka, Japan; University of Sydney, AUSTRALIA

## Abstract

During the past 100 years in densely populated south-eastern Brazil, wetlands have been severely transformed due to urbanization, agriculture and mining. The recently discovered São Paulo Marsh Antwren (*Formicivora paludicola*) is endemic to these wetlands, and is listed as “Critically Endangered” by the IUCN. The species is only found in an area of 1.42 km^2^, it has a sparse and fragmented distribution, low dispersal capacity, and has probably lost around 300 km^2^ of habitat in the past 100 years. Furthermore, very little is known about *F*. *paludicola* natural history, and so it is difficult to construct a robust conservation plan. Using Kernel home range estimations and the Adjusted-SD/Torus Shift test (a novel tool for animal-habitat association studies), we showed that the species avoids patches of the alien invasive ginger lily (*Hedychium coronarium*). Given the high density of their population (3.6 mature individuals/ha), *F*. *paludicola* could thrive in relatively small areas of suitable wetlands protected from human occupation and water contamination, however special attention should be paid to biological invasions, which may represent a serious threat to the remaining populations. Protecting a few small wetlands used by *F*. *paludicola* would be an important step towards general conservation and restoration of Atlantic Forest wetlands and its endemic endangered species.

## Introduction

The Brazilian Atlantic Rainforest is a biodiversity hotspot, and one of the most endangered ecosystems in the world [1, 2]. Although the Atlantic Forest primarily comprises forest habitats, wetlands also play an important and largely neglected role in the region’s ecology. In Brazil’s south-eastern Atlantic Forest, at the Upper Tietê and Upper Paraíba do Sul basins, more than 400 km^2^ of marshes and wetlands have been lost due to human activities [3]. The abundant water and level ground made this area very attractive to human occupation [3, 4] so that today, pastures, crops, mining, cities and industries dominate a landscape once covered by vast wetlands. Human development has also resulted in the large-scale pollution of regional water as well as changes in flow due to the construction of dams and reservoirs [5]. Nevertheless, the São Paulo Marsh Antwren (*Formicivora paludicola*—Thamnophilidae) managed to survive in this region and remained unknown to science until its discovery in 2005 and description in 2014 [6].

The distribution of *F*. *paludicola* is limited to fragmented wetlands in the Atlantic Forest, and it is faced with continuous habitat reduction. Recent estimates show that this species could have lost more than 300 km^2^ of suitable habitat within its range within the last 100 years [7]. Today, *F*. *paludicola* is considered one of the most threatened Thamnophilids in the world (“Critically Endangered” according to IUCN criteria; [8]), mostly because it has a total area of occupancy of only 1.42 km^2^ [7]. This is extremely small when compared to other members of the family [9]. It also has limited dispersal ability (average 25 m; [10]) and narrow habitat specificity, as it has never been recorded in habitat other than marshes composed of cattail (*Typha dominguensis*), giant bulrush (*Schoenoplectus californicus*) or switchgrass (*Panicum* sp.). These marshes have been reduced by more than 90% over the last 100 years [7].

As with most Thamnophilids, *F*. *paludicola* is territorial and socially monogamous [11]. The dispersal pattern of territorial individuals thus tends to be regular and evenly spaced [12] with pairs defending territories throughout the year [13–18]. Understanding these population spatial patterns can provide important insight into distribution, abundance, dispersion, and even speciation processes underlying current occurrence [19, 20]. Home range (the area crossed by an individual in its habitual activities; foraging, mating and parental behaviour, excluding environments only rarely entered into; [21]) studies can also contribute to the comprehension of the relationship between an animal and its environment. This information has an important role in the delimitation of protected areas for a species’ conservation, and can be used in evaluations of habitat preferences [22, 23]. Habitat use is considered selective when an animal uses particular types of habitats disproportionally more than others, instead of using all the available habitats randomly [24]. The description of a species’ habitat use is thus highly relevant to understanding its ecological relationships, foraging and nesting strategies, and in identifying the most appropriate areas to be protected [25].

There has been growing recognition regarding the importance of incorporating both habitat and demographic information into conservation planning [26]. Besides our recent efforts to study the conservation status of *F*. *paludicola* based on its distribution, occupancy models, and population genetics [7, 11, 27], no formal study has described this species’ home range, density, natural history, and habitat association. This lack of data prevents a robust conservation initiative.

Therefore, to meet the need for a more detailed understanding of its natural history, in this paper we address home range and habitat association data for *F*. *paludicola*. We show relevant features that can predict its spatial use, using a novel tool, the Adjusted-SD/Torus Shif test [28, 29]. We describe *F*. *paludicola* habitat preferences and discuss the fitness consequences associated with the avoidance of an exotic invasive plant, the ginger lily [30]. Supported by this data, we suggest which are the most adequate areas to be preserved, or to receive the birds in the case of translocation efforts (as suggested by [27]). Such actions would greatly benefit the conservation of the Atlantic Forest wetlands—an important reservoir in a scenario of recurrent water supply crisis.

## Methods

### Study sites

**Fig 1:**
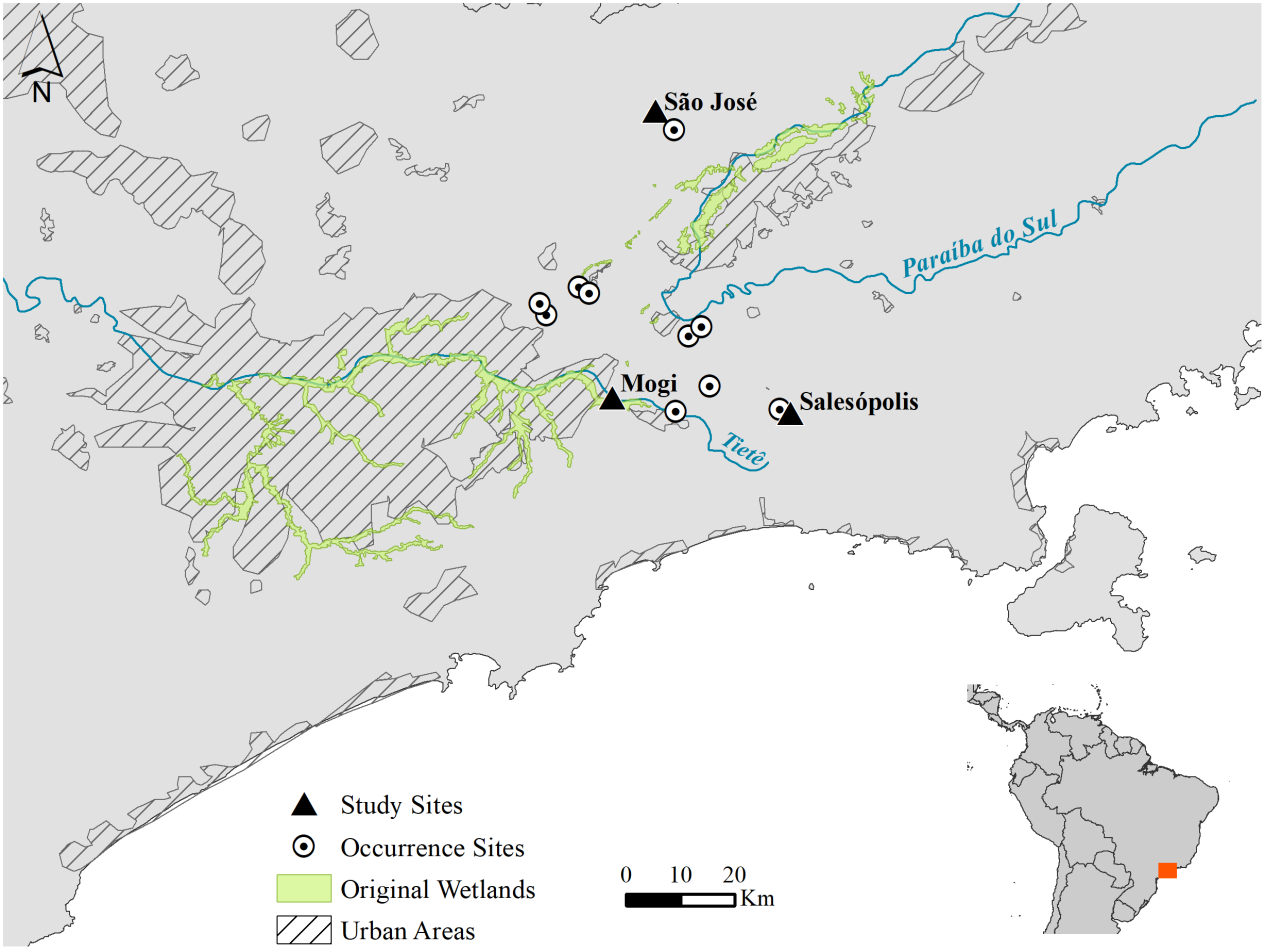
Occurrence of *Formicivora paludicola*. Circles and triangles indicate marshes where São Paulo Marsh Antwren (*Formicivora paludicola*) is found at Upper Tietê Basin and Upper Paraíba do Sul Basin (south-eastern Brasil). Triangles indicate the three sites where we examined animal-habitat association. Modified from [27].

Data collection was carried out in the three largest marshes occupied by *F*. *paludicola* (annual mean temperature 18.1°C; annual mean rainfall is 1491 mm [31]). Mogi Marsh (hereafter Mogi; 23°32’45”S 46°07’14”W; elevation: 688 m; ~23 ha) is located in the city of Mogi das Cruzes. This falls within the Upper Tietê Basin and the vegetation is predominantly composed of cattail (*Typha dominguensis*) and surrounded by a secondary-growth forest matrix. The second site, São José Marsh (hereafter São José; 23°04’15”S/46°02’44”W; elevation: 598 m; ~1.56 ha) is in the city of São José dos Campos. The marsh vegetation is predominantly composed of cattail and ginger lily (*Hedychium coronarium*), and the site is surrounded by *Eucalyptus* plantations. The third site, Salesópolis Marsh (hereafter Salesópolis; 23°34’26”S/45°49’24”W; elevation: 866 m; ~5.3ha) is located near the city of Salesópolis, only 8 km away from the headwaters of the Tietê River. The vegetation is composed primarily of cattail, giant bulrush, switchgrass and saw-sedge (*Cladium mariscus*), and surrounded by secondary forest growth and *Eucalyptus* plantations (Fig 1).

### Capturing and banding

During 55 days (440 net hours) of field work, we banded 53 individuals. The time spent at each site was proportional to its size; we spent 30 days at Mogi, 15 days at Salesópolis, and 12 days at São José. We walked in predefined transects (as explained below) playing a standardized sequence of *F*. *paludicola* songs and calls. When unbanded individuals were sighted, we set up a 12 m mist net and used playbacks to attempt to to capture the birds. All birds captured were banded with uniquely numbered bands supplied by the Brazilian National Banding System (Centro de Pesquisa para Conservação das Aves Silvestres), and a unique combination of colored bands. The banding procedures followed The North American Bander’s Study Guide [32], and the Brazilian Manual of Banding [33]. Banded individuals were released at the site of capture.

### Monitoring

For one year (August 2012-August 2013), we monitored the banded birds. We visited each marsh on a monthly basis and walked predefined transects (1 m wide) at a speed of 0.01 km/h. The transects were set 35 m apart and ran parallel to each other across the full length of each marsh (4 trails 500 m long in Mogi, 3 trails 300 m long in Salesópolis and 4 trails 100 m long in São José dos Campos). We spent a total of 620 h in Mogi (103 days), 336 h in Salesópolis (56 days) and 108 h in São José (26 days) divided equally between months. Every three months we moved the trail by 10 m to either side, depending on the local land features, to avoid spatial bias in our data. We sampled a total of 16 transects in Mogi, 12 in Salesópolis and 16 in São José. When a banded individual was sighted, we identified it and noted its geographical coordinates using a hand-held Global Position System unit (Garmin GPSMAP 62s). The GPS location error was usually lower than 4 m. When we heard a bird vocalize, if less than 15 m away, we used playback to call the bird for identification. When using playback, we noted only the initial position of the bird on that day, to avoid problems with the influence of playback on its behaviour [34].

### Home range estimations

Using the adehabitatHR package [35] in R software 3.0.2 [36], we applied the Fixed-Kernel method to calculate home ranges (95% of utilization distribution) and home range overlap. This method is one of the most reliable to estimate home ranges [37, 38] and measures the intensity of area use, while excluding areas that are rarely used by the birds [39]. To calculate the smoothing parameter, we used the Least Square Cross Validation approach [37]. We also calculated home range sizes using 95% minimum convex polygons (MCP). Although it is a frequently used method for determining range size from a sample of locations [40], the minimum convex polygon does not describe the intensity with which an animal uses different parts of its home range [41]. This method is also strongly influenced by outlying locations [42]. Thus, we limited our use of this method to comparisons of home range size among Thamnophilids [10, 15, 43–46]. Although few Thamnophilids have been studied using the Fixed-Kernel method [47], we preferred Kernel methods for estimating the home range size of *F*. *paludicola*, given the intrinsic problems associated with MCP methods [39, 41, 48–54].

MCP area/number of observation plots reached an asymptote when birds were present at 25 or fewer locations. Therefore, we excluded birds with fewer than 25 locations from our analysis, leaving us with 15 of the 53 banded individuals (Mogi = 7; São José = 4; Salesópolis = 4). As a standard rule, we considered that the curve had reached stability when five additional observations did not provide an increase in area greater than one percent (modified from [55, 56]).

### Habitat association

Aggregated distributions, such as the distribution resulting from territoriality, imply spatial autocorrelation. Most techniques available for animal/plants-habitat association studies do not deal well with spatial autocorrelation [29]. Most statistical methods fail to consider that an individual’s use of a particular habitat affects its use of other habitats [57, 58]. A possible solution is the use of analytical techniques that test for departures from random use of habitat features, taking spatial autocorrelation in consideration. If non-random use of a specific habitat feature is detected, other techniques should be applied to determine which habitats are used more or less than expected purely by chance [58, 59]. In this study, we used an Adjusted-SD test combined with a Torus Shift (S1 Appendix), where an original rectangular habitat map is shifted randomly on a two-dimensional torus [28, 29]. This shift retains the overall autocorrelated patterns (home range use); therefore, the expected density of points is the same at any habitat due to the Torus Shift, creating a number of null scenarios. The null hypothesis was that the observed relationship between habitat and *F*. *paludicola* distribution is consistent with the stationary distribution model, which generates spatial patterns influenced by factors independent of habitat [29]. We applied a Torus Shift to the habitat maps of the three study sites, while retaining the density estimates of 15 individuals of *F*. *paludicola* obtained by the Fixed-Kernel method.

**Fig 2:**
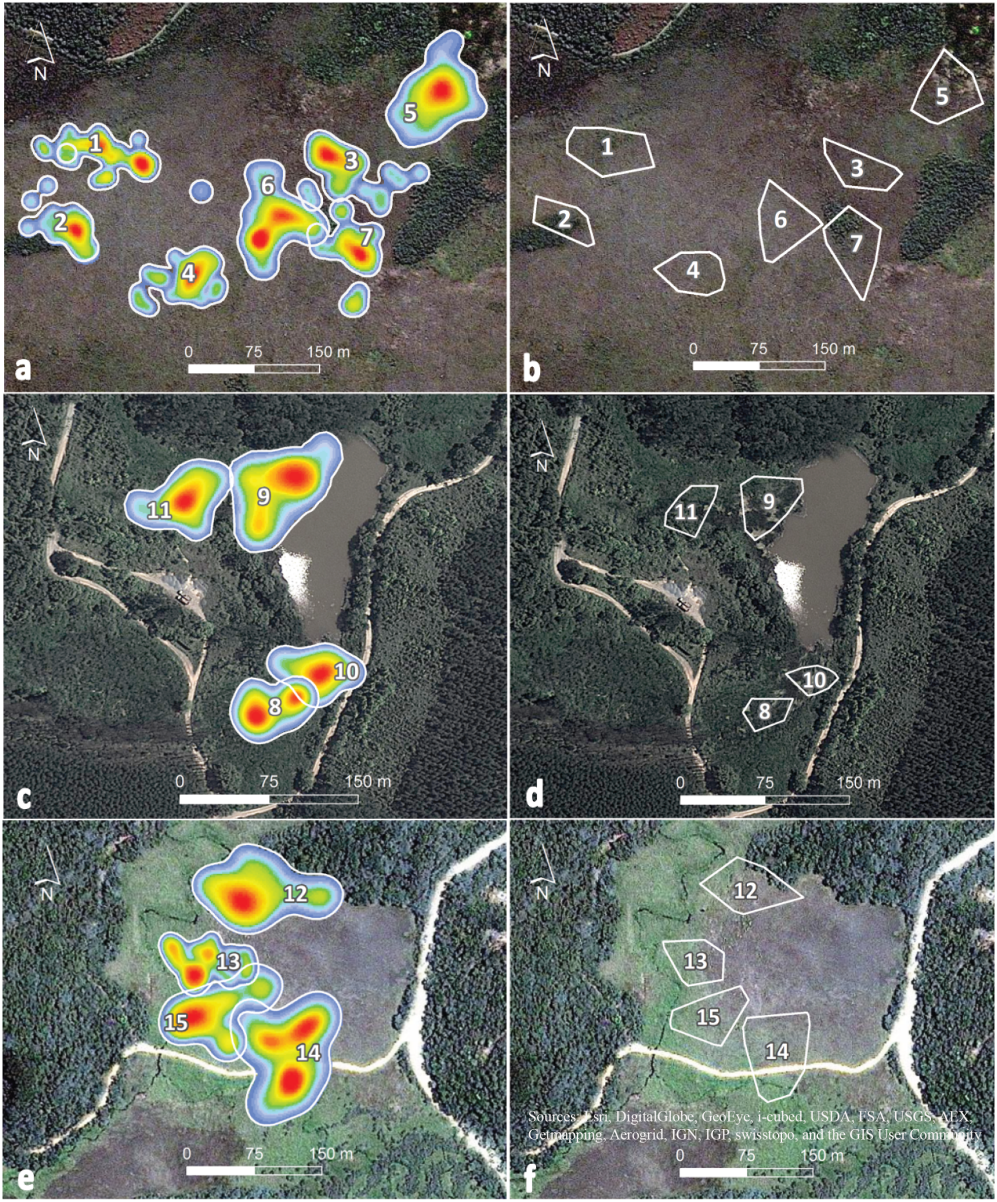
Home-range of *Formicivora paludicola*. Kernel home-range estimates for (**a**) Mogi, (**c**) São José and (**e**) Salesópolis marshes, respectively and (**b**, **d**, **f**) 95% Minimum Convex Polygons obtained for the same sites. Numbers indicate individual birds (Table 1). World Imagery—Source: Esri, DigitalGlobe, GeoEye, Earthstar Geographics, CNES/Airbus DS, USDA, USGS, AEX, Getmapping, Aerogrid, IGN, IGP, swisstopo, and the GIS User Community. Map image is the intellectual property of Esri and is used herein under license. Copyright © 2017 Esri and its licensors. All rights reserved.

The first step to constructing the habitat map for the Torus Shift simulations involved overlaying a grid of 35 x 35 m plots on each marsh. At the intersection of each plot, we measured habitat features in 1 m^2^ quadrats (the number of quadrats was proportional to the size of the site). The total size of each grid was 15.1 ha in Mogi, 4.3 ha in Salesópolis and 2.85 ha in São José dos Campos. We collected data on the average cattail height (in mm; by *sward stick*; [60, 61]), cattail density (by counting individuals in the 1 m^2^ quadrats; [62]), vegetation cover (by visual estimation in the 1 m^2^ quadrats, [62]) and water depth (in cm; getting an average measurement for two different seasons, using water depth stocks). In Salesópolis, we replaced cattail height for giant bulrush (*Schoenoplectus californicus*) height (also measured by *sward stick*; [60, 61]) and added giant bulrush density to our analysis. Data obtained along a dirt road that divides the home range of one of the individuals monitored (Fig 2E) were excluded from our analysis. In São José, we also measured ginger lily (*Hedychium coronarium*) density (by counting individuals in 1 m^2^ allotments), as this invasive plant occurs in dense stands at this site. In order to avoid subjectivity in the definition of habitat categories, we worked with continuous habitat variables [63, 64]. Once we collected the habitat data, the second step in continuous habitat map construction was the interpolation method of Ordinary Kriging in the ‘kriging’ package in R software (‘spherical model’, lags = 5) [65]. At this step, we divided the São José site in two subsets (São José 1 and São José 2) to not interpolate vegetation features over a lake separating both areas. The map was shifted along the x and y coordinates by 5 m steps on a two-dimensional torus, with the number of combinations (number of null models generated) dependent on the size of the site (Mogi = 24,156 shifts; São José = 4,516 shifts; Salesópolis = 6,880 shifts). We also used mirror and rotated maps for each shifted image [28, 29].

To check for association between the *F*. *paludicola* density estimates and these habitat features, we calculated the SD of *p(E|x)* (conditional probability of observation of marked birds given a habitat variable *x*) for all the shifted maps, and used it as the statistic for the significance test. We considered the species associated to an environmental feature if the observed SD was within the lower 5% of values obtained by the Torus simulation (random chance). A small SD of *p(E|x)* is expected for a species associated with a few specific habitat features, because a habitat specialist has a higher *p(E|x)* within a certain range of *x* than in other habitats [29]. We used functions of an Adjusted-SD test to access the range of habitat features associated with the species [29].

Field work was undertaken in accordance with Brazilian laws (permit number: 31224 Ministério do Meio Ambiente/ Instituto Chico Mendes de Conservação da Biodiversidade/ Sistema de Autorização e Informação em Biodiversidade). Areas accessed were privately owned, and we did not sample protected areas. The owners of the lands (Suzano Papel e Celulose, and small agricultural producers) gave permission to conduct the study on each site. Animal manipulation was undertaken under approval of Conselho Nacional da Experimentação Animal (Comissão de Ética do Uso Animal—28198–5).

## Results

### Home range

We used 452 locations of 15 individuals for home range estimations. To avoid lack of convergence in the models (which happens when both LSCV is used for bandwidth choice, and a high number of points cluster together), we used up to 32 locations per individual in our analysis (S1 Dataset).

The largest 95% Kernel home range (1.07 ha) was almost six times larger than the smallest (0.18 ha) (Table 1; Fig 2), although, in the general, the home range estimates were not very variable (Q_1_ = 0.42 ha and Q_3_ = 0.71 ha). The average value of 95% Kernel home range size for *F*. *paludicola* was 0.55 ha (SD = 0.24) (Table 1).

**Table 1:** *Formicivora paludicola* home range estimates.

	Individual Identification	Number of Relocations	Sex	Kernel UD (ha)	MCP area (ha)
**Mogi**	D92460 (1)	32	F	0.52	0.35
	D92439 (2)	32	M	0.39	0.19
	D92453 (3)	31	F	0.55	0.24
	D92457 (4)	32	M	0.49	0.24
	D92443 (5)	32	M	0.75	0.33
	D92448 (6)	30	M	0.73	0.32
	E130230 (7)	32	F	0.52	0.33
**average**		-	-	**0.56±0.13**	**0.28±0.06**
**São José**	E130215 (8)	32	F	0.18	0.07
	E130219 (9)	32	F	0.52	0.17
	E130216 (10)	31	M	0.19	0.06
	E130221 (11)	32	M	0.33	0.11
**average**		-	-	**0.30±0.15**	**0.10±0.05**
**Salesópolis**	E130208 (12)	31	M	0.88	0.33
	E130206 (13)	31	F	0.45	0.21
	E130201 (14)	32	M	1.07	0.47
	E130204 (15)	32	M	0.70	0.28
**average**		-	-	**0.77±0.26**	**0.32±0.10**
**Total average**		-	-	**0.55±0.24**	**0.25±0.11**

Home range estimates obtained by Kernel and minimum convex polygon (MCP) methods, for 15 individuals of *F*. *paludicola* in three sites (Mogi, São José and Salesópolis). “Individual identification” refers to the ring number of each banded animal, the numbers brackets are the numbers in Fig 2.

The largest MCP value estimated was 0.47 ha while the smallest was 0.06 ha (Table 1; Fig 2). The average MCP value was 0.25 ha (SD = 0.11), and variance was low (Q_1_ = 0.18 ha and Q_3_ = 0.33 ha). Home range overlap between adjacent individuals averaged 40% (SD = 27%) at Mogi; 57% (SD = 0.26) at Salesópolis; and 67% (SD = 0.27) at São José.

### Habitat association

The probability of finding *F*. *paludicola* was correlated with cattail height (Adjusted-SD test; SD_1_<0.01; SD_2_<0.01), cattail density (SD_1_ = 0.04; SD_2_ = 0.03), water depth (SD_1_ = 0.03; SD_2_ = 0.03), and ginger lily density (SD_1_<0.01); SD_2_<0.01) in both subsets of São José (S2 Dataset). At Salesópolis, *F*. *paludicola* occurrence was correlated with giant bulrush height (observed SD<0.01). At Mogi, the probability of finding *F*. *paludicola* was independent of our habitat measurements (Table 2).

**Table 2:** Adjusted-SD results.

Marsh	cattail height	cattail density	Plant cover	water depth	giant bulrush density	giant bulrush height	ginger lily density
Mogi	0.79	0.11	0.93	0.99	-	-	-
São José 1	**< 0.01**^b^	**0.04**^b^	0.24	**0.03**^b^	-	-	**< 0.01**^a^
São José 2	**< 0.01**^b^	**0.03**^b^	0.1	**0.03**^b^	-	-	**< 0.01**^a^
Salesópolis	-	0.64	0.07	0.9	0.86	**0.01**	-

We considered that *F*. *paludicola* is associated with the habitat variable, when the observed SD of *p(E|x)* (conditional probability of observation of marked birds given a habitat variable *x*) was within the smallest 5% (0.05) SD values obtained by Torus simulation.

^a^negative association

^b^positive association

**Fig 3:**
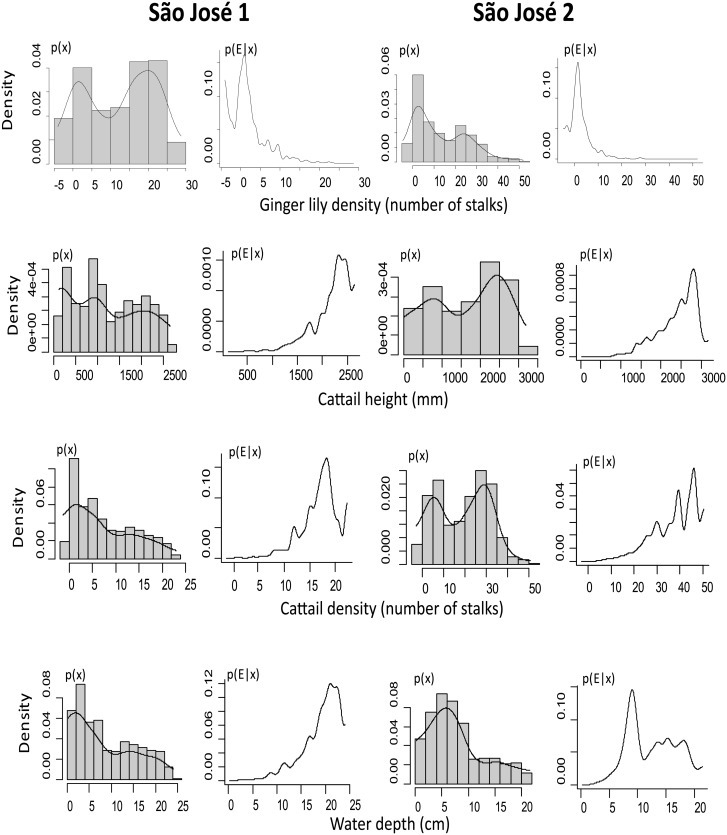
Density functions of habitat association in São José. Estimated density functions of Adjusted-SD test along a habitat gradient showing association between *F*. *paludicola* and four habitat variables in two subsets of São José. *p(x)* is the probability that a selected point is within habitat *“x”*, then, these graphs represent how abundant a habitat feature is in the study plot. *p(E*|*x)* is the conditional probability of *F*. *paludicola* being observed at any selected point that has habitat feature “*x*”. The single peak in the *p(E*|*x)* indicates a strong association (positive or negative) between the presence of the bird with the habitat variable.

In both subsets of São José, the probability density of *F*. *paludicola* was negatively associated with ginger lily density, and positively with cattail height, cattail density, and water depth (Fig 3). At Salesópolis, the same graphs point to the polarization of *p(E|x)* within higher values of giant bulrush height. At Mogi again, none of the measured variables were important (S2 Appendix).

## Discussion

Here we provide the first studies on home range and habitat preferences of *F*. *paludicola*. For the first time, we report that an invasive plant might threaten this species. Adjusted-SD/Torus Shift test showed that *F*. *paludicola* avoids patches densely inhabited by the exotic ginger lily. Biological invasions are considered one of the main causes of loss in biological diversity, and one of the greatest negative environmental impacts caused by humans [66–68]. Ginger lily was brought to South America from Asia as an ornamental plant, and it has a high capacity for propagation and rapid growth [69]. Ginger lily growth causes shading of native herbs and seedlings, affecting plant community structure, and rapidly decreasing plant diversity [70–72].

Our results suggest that ginger lily stands might not be optimal for *F*. *paludicola* occurrence. Suboptimal areas could, however, be important for the maintenance of population dynamics. For instance, under the source-sink dynamics model, a “sink” area would be a low-quality habitat where breeding success is insufficient to balance local mortality [73, 74]. On the other hand, “source” habitats are high quality areas where the populations can increase. The excess individuals from source areas would eventually occupy sink habitats, at least during part of their lives [74]. The use of sink areas by part of the population, with possible back-dispersal to sources, could lead to the maintenance of larger populations than in areas where only source habitats would be available [73]. Ginger lily stands could be acting as sink areas for *F*. *paludicola*, yet we did not measure habitat use of floater individuals—those without established territories—for which ginger lily stands could be habitable. In general, further studies of population dynamics, considering birth, death, immigration and emigration rates are necessary to understand if habitat use of *F*. *paludicola* is consistent with source-sink dynamics [74]. For now, our results support the idea that ginger lily is avoided by monogamous pairs of birds with established territories. Hence, ginger lily avoidance could indicate that there is selection against individuals trying to establish territories in ginger lily stands [75]. It is possible then that ginger lily has a negative impact on the survivorship, and reproductive potential of pairs of *F*. *paludicola*.

Invasion of exotic plant species has been reported to affect birds’ survivorship by increasing nest predation [76], and decreasing food availability [77]. Here we did not measure effects of ginger lily on *F*. *paludicola* survivorship, although the correlation between ginger lily density and water depth might provide some clues of how *F*. *paludicola* habitat quality would be affected by the presence of this invasive plant. In this study, we found a relationship between *F*. *paludicola* occurrence and water depth only in the marsh invaded by ginger lily. Ginger lily’s rapid growth might obstruct water flow in wetlands and creeks, which affects the maintenance of regular water cycles [71, 78]. Based on [71, 78], we suggest that there is a potential negative effect of ginger lily growth on water depth in our study site. In fact, ginger lily rhizomes are less tolerant of hypoxia and anoxia than native wetland plants rhizomes [79]. Since *F*. *paludicola* collects most of its food items (arthropods) from the water surface [6, 80], the bird would have fewer opportunities to prey on its preferred food sources in the drier ginger lily stands. Although we attempted to quantify aquatic arthropods abundance inside and outside ginger lily patches, our traps failed to collect arthropods in a standardized manner. We suggest that further investigation is necessary to clarify whether ginger lily in our study sites affects water levels, food availability, and survivorship of *F*. *paludicola*.

At Salesópolis, where the dominant vegetation consists of giant bulrush instead of cattail, we found the largest Kernel home range sizes (average of 0.77 ha). The original habitats of *F*. *paludicola* were likely marshes dominated by giant bulrush, although the species should have adapted to cattail habitats secondarily [6]. At São José, which is the smallest study site, and the only one occupied by ginger lily, we got the smallest average home range (0.30 ha), and the highest percentage of home range overlap. Because we only compared home range size in three different sites, we do not discuss possible relationships between species density, marsh size and habitat quality [81].

MCP estimates obtained for *F*. *paludicola* are the same as obtained for *F*. *acutirostris* in tidal marshes, an average territory size of 0.25 ha [10]. These are the smallest territories measured for Thamnophilids. In Amazonian forest the smallest Thamnophilid territories belong to the bamboo specialist *Cercomacra manu* (0.6 ha) and to *Cercomacra tyranina* (0.3 ha) [15, 43, 44]. In Atlantic Forest, the smallest territory sizes measured, besides those of *F*. *acutirostris* and *F*. *paludicola*, belong to *Dysithamnus mentalis* (0.7 ha) and *Drymophila ferruginea* (0.6 ha) [45, 46].

Kernel home range estimates revealed that *F*. *paludicola* occurs in high densities in the three study sites when compared to other Tropical Passerine species. Average density values across the three study sites are 3.6 mature individuals/ha. Within Thamnophilidae, this number is only surpassed by the Amazonian *Cercomacra tyrannina* (6.67 mature individuals/ha; [82]) and *Cercomacra manu* (4–10 mature individuals/ha; [17]). Estimates of number of individuals per marsh based on marsh size are consistent with effective population sizes obtained with population genetics data: 99.9 individuals in Mogi, 28.8 individuals in Salesópolis, and 34.4 individuals in São José [27]. The reduced size of the populations in three of the mains areas inhabited by *F*. *paludicola* emphasizes the necessity of conservation actions to protect this species. However, the fact that *F*. *paludicola* lives in high densities in the few marshes it occupies highlights how valuable relatively small patches of marsh could be for the conservation of this species.

*Formicivora paludicola* is found in a small region inhabited by more than 16 million people (Upper Tietê Basin, Upper Paraíba do Sul Basin) [83]. This huge human presence is reflected in complex forms of occupancy and use of natural resources [84]. Marshes are extremely vulnerable to the expansion of areas dedicated to agriculture, livestock grazing, mining, industries and residential areas. Furthermore, between 2014 and 2015, southeastern Brazil experienced one of the most severe droughts in decades. Warmer temperatures and rainfall deficiency led to a water supply crisis, which was aggravated by increasing population and water consumption [85]. Further studies are necessary to indicate if the water crisis was generated by human induced changes in temperature and precipitation [85], and which will be the impacts of climatic changes on *F*. *paludicola* populations.

In a situation of such extreme fragmentation, habitat loss, and climatic changes, we strongly advise the creation of a network of protected areas connected by restored wetlands in Alto Tietê and Alto Paraíba do Sul regions. With such a network, we believe that the extant populations could thrive and colonize new areas. These areas should also be protected from water contamination and especially from the invasion of exotic plant species such as the ginger lily. This situation is comparable to the main threat faced by the “Endangered” *F*. *acutirostris*. In southern Brazil, the biological invasion of tidal marshes by exotic grass species such as *Urochloa arrecta* and *Brachiaria mutica* affects nest construction and breeding success of *F*. *acutirostris* [10].

We emphasize that Mogi and Salesópolis marshes should be provided the most protection, because they contain the most numerous populations of *F*. *paludicola* and have not suffered from extensive invasion by exotic plants. In between these two sites is another population that deserves special attention, inhabiting an area that is presently being drained by sand mining companies. This population offers a means of restoring connectivity between Mogi and Salesópolis marshes, if some restoration efforts are carried out in the rural areas in between. We also urge the formation of management strategies to control the spread of exotic plants, especially in São José, which has the largest *F*. *paludicola* population in the Paraíba do Sul Basin. The creation and management of these areas, with constant monitoring of fauna and flora, would ensure continued growth in our understanding of regional bird dispersion, diversification, and ecology. In addition, it would help to conserve a remnant of the nearly extirpated Atlantic Forest wetlands, and to protect many of São Paulo’s additional threatened wetland species, such as the Lesser Grass-Finch (*Emberizoides ypiranganus*), and Long-winged Harrier (*Circus buffoni*) [86]. It would also conserve Upper Tietê and/or Upper Paraíba do Sul Basin endemic fish species: *Hyphessobrycon duragenys*, *Hyphessobrycon flammeus*, *Spintherobolus papilliferus*, *Heptapterus multiradiatus*, *Pseudotocinclus parahybae*, and the amphibian *Cycloramphus semipalmatus* [87, 88].

For the first time in zoological studies, the Adjusted-SD/Torus Shift test was used to indicate an animal’s habitat preference. Our results indicate that ginger lily is avoided by *F*. *paludicola*, suggesting that biological invasions might represent an additional, never before reported threat to this “Critically Endangered” species. *Formicivora paludicola* provides an impetus for the creation of a network of protected wetlands, not invaded by ginger lily, in the Upper Tietê Basin. Besides providing adequate habitat for the maintenance of populations of *F*. *paludicola*, a restored Atlantic Forest wetland network would also help to preserve the state of São Paulo’s water quality, water supply, recharge groundwater, and to mitigate floods. Ultimately, we suggest the Adjusted-SD/Torus Shift test as a useful method for animal-habitat association studies, which could be employed to assess habitat preferences of threatened (or other) animal species worldwide.

## Supporting information

S1 Dataset Points used for home range estimates. Geographic position (UTM) of each bird individual used for Minimum Convex Polygon and Kernel home range estimation. Birds’ ID refer to Table 1.
(CSV)

S2 Dataset Environmental information plugged in Adjusted-SD analysis. Data on vegetation height, coverage, density and water height.
(CSV)

S1 Appendix R script used to perform Adjusted-SD/Torus Shift analysis. (TXT)

S2 Appendix Estimated density functions of Adjusted-SD test along a habitat gradient in Mogi and Salesópolis marshes. Functions of Adjusted-SD test for Mogi and Salesópolis sites. *p(x)* is the probability that a random point selected is within a habitat “*x*”. *p(E|x)* is the probability of São Paulo Marsh Antwren (*F*. *paludicola*) occurring at any selected point that has habitat feature “*x*”.
(TIF)
