# Supplementary figures and images for: Plant invasion: Another threat to the São Paulo Marsh Antwren (Formicivora paludicola), a species on the verge of extinction
Source: PLoS One. 2017 Dec 27;12(12):e0189465. doi: 10.1371/journal.pone.0189465 (PMC5744942; doi:10.1371/journal.pone.0189465)

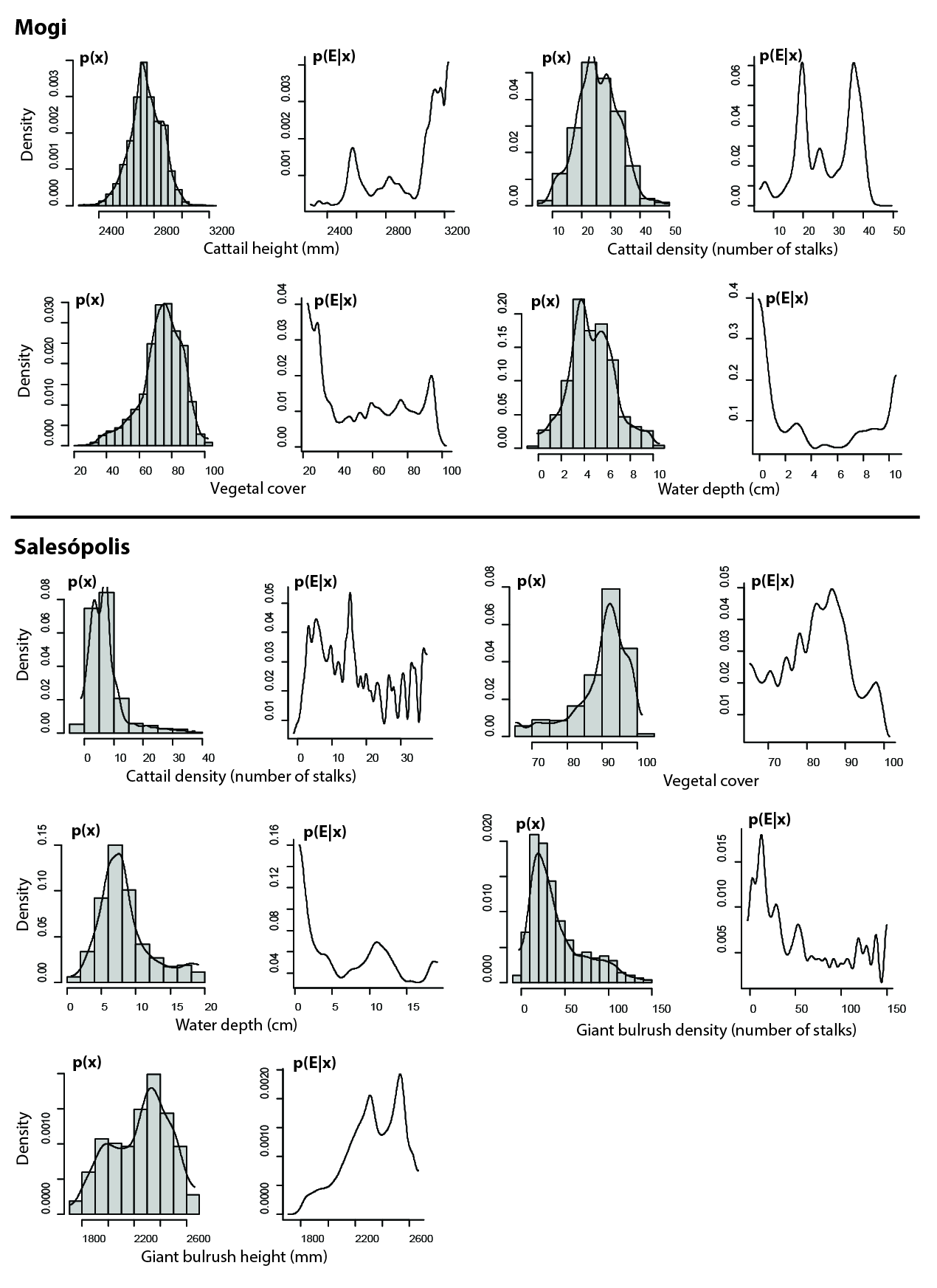

Supplement: S2 Appendix — Functions of Adjusted-SD test for Mogi and Salesópolis sites. p(x) is the probability that a random point selected is within a habitat “x”. p(E|x) is the probability of São Paulo Marsh Antwren (F. paludicola) occurring at any selected point that has habitat feature “x”. (TIF) [file pone.0189465.s004.tif]
